# Temporal Dynamics and Genomic Landscape of SARS-CoV-2 After Four Years of Evolution

**DOI:** 10.7759/cureus.53654

**Published:** 2024-02-05

**Authors:** Abdelmounim Essabbar, Safae El Mazouri, Nassma Boumajdi, Houda Bendani, Tarik Aanniz, Ouadghiri Mouna, Belyamani Lahcen, Azeddine Ibrahimi

**Affiliations:** 1 Biotechnology Lab (MedBiotech) Bioinova Research Center, Rabat Medical & Pharmacy School, Mohammed V University, Rabat, MAR; 2 Toulouse Cancer Research Center, Institut National de la Santé et de la Recherche Médicale (INSERM), Toulouse, FRA; 3 Émergency Department, Military Hospital Rabat Morocco, Rabat, MAR; 4 Mohammed VI Center For Research and Innovation, Mohammed VI University of Sciences and Health, Rabat, MAR

**Keywords:** recombinant variants, viral evolution, genomic surveillance, lineages, mutations, covid-19, sars-cov-2

## Abstract

Introduction

Since its emergence, severe acute respiratory syndrome coronavirus 2 (SARS-CoV-2) has undergone extensive genomic evolution, impacting public health policies, diagnosis, medication, and vaccine development. This study leverages advanced bioinformatics to assess the virus’s temporal and regional genomic evolution from December 2019 to October 2023.

Methods

Our analysis incorporates 16,575 complete SARS-CoV-2 sequences collected from 214 countries. These samples were comparatively analyzed, with a detailed characterization of nucleic mutations, lineages, distribution, and evolutionary patterns during each year, using the Wuhan-Hu-1 strain as the reference.

Results

Our analysis has identified a total of 21,580 mutations that we classified into transient mutations, which diminished over time, and persistent mutations with steadily increasing frequencies. This mutation landscape led to a notable surge in the evolutionary rate, rising from 13 mutations per sample in 2020 to 96 by 2023, with minor geographic variations. The phylogenetic analysis unveiled three distinct evolutionary branches, each representing unique viral evolution pathways. These lineages exhibited a tendency for a reduced duration of dominance with a shortening prevalence period over time, as dominant strains were consistently replaced by more fit variants. Notably, the emergence of the Alpha and Delta variants in 2021 was followed by the subsequent dominance of Omicron clade variants that have branched into several recombinant variants in 2022, marking a significant shift in the viral landscape.

Conclusion

This study sheds light on the dynamic nature of SARS-CoV-2 evolution, emphasizing the importance of continuous and vigilant genomic surveillance. The dominance of recombinant lineages, coupled with the disappearance of local variants, underscores the virus’s adaptability.

## Introduction

In the last four years, severe acute respiratory syndrome coronavirus 2 (SARS-CoV-2) has caused over 700 million infections leading to 7 million deaths globally: in 2023, 38 million infections and nearly 288,000 deaths were recorded [1]. This alarming toll has urged collaborative efforts within scientific and research communities to deepen our understanding of the virus and the associated disease. Despite dedicated endeavors, the virus’s relentless evolution continues to give rise to novel variants with distinct characteristics [2]. The emergence of SARS-CoV-2 mutations over the past four years has been a dynamic process influenced by factors such as random molecular-scale shifts, replication errors, and the selective pressure exerted by the host immune response. This ongoing evolution underscores the virus’s ability to adapt and persist in the face of changing environmental and host conditions. Previous research has unveiled over 50,000 nucleotide variations, exhibiting uneven distribution across the 29 genes that constitute the SARS-CoV-2 genome, notably in key hotspots such as those located in the receptor-binding domains (RBD), driving the virus’s evolution [2,3]. These hotspot mutations have displayed a significant impact on the virus’s ability to bind to host cells, contributing to its increased activity or infectivity over time [3].

Since the early stages of the pandemic, the initial mutation, D614G, significantly impacted the spike protein, leading to heightened transmissibility on a global scale [2]. Then, the Q677P/H mutations gained prominence in late 2020, underscoring the virus’s adaptability [3,4]. Subsequently, the A701V mutation surfaced in December 2020, raising uncertainties regarding its infectivity and aggressiveness [5]. In January 2021, the P681H mutation, linked to the Alpha variant, demonstrated a notable surge in global frequency, echoing the trajectory of the now-prevalent D614G mutation [5]. Progressing further, the N501Y mutation, associated with increased binding affinity, became prevalent in variants such as Alpha, Beta, and Gamma [6]. Additionally, by mid-2021, mutations within the RBD, including S477G/N, Y453F, L452R, G446V, and N440K, were reported to enhance viral binding to the human angiotensin-converting enzyme 2 (hACE2) receptor, exerting an influence on vaccine design considerations [5].

These mutations also held potential associations with increased SARS-CoV-2 transmission in specific regions like India and Japan [7]. As these mutations have shaped the genomic landscape of SARS-CoV-2, they have given rise to diverse lineages, each characterized by specific genetic signatures and evolutionary trajectories. Notably, certain lineages have exhibited increased transmissibility, altered antigenic properties, and impacted disease severity [4,8]. The evolutionary trajectory of SARS-CoV-2 began with the original strains, characterized by the prototypic genomic makeup of the virus. Subsequently, the emergence of clades 19A, 19B, and 20B marked early stages of genetic diversification, laying the foundation for subsequent lineages. Clade 19A exhibited distinct genetic features, contributing to the virus’s adaptive changes over time. Clade 19B continued this trend, demonstrating incremental genetic shifts that influenced the virus’s transmissibility and host interactions. Subsequently, after the first year, the Alpha variant (Lineage B.1.1.7) emerged in the United Kingdom, demonstrating increased transmissibility and rapid global spread [8,9]. Simultaneously, the Beta variant (Lineage B.1.351) was identified in South Africa, characterized by mutations that influenced virus-host interactions and immune evasion, marking significant shifts in the genomic landscape. Both variants represented distinct characteristics in terms of transmissibility and immune response, with Alpha exhibiting heightened transmissibility and Beta associated with increased severity and mutations enhancing binding affinity [4,6]. As the pandemic progressed, more virulent variants surfaced. The Gamma variant (Lineage P.1), first emerging in Brazil, was accompanied by the Delta variant (Lineage B.1.617.2) in India [7]. The Gamma variant exhibited heightened transmissibility and the potential for immune evasion, while the Delta variant displayed increased transmissibility and achieved global dominance by mid-2021. In early 2022, a new set of variants, collectively known as the Omicron variants (clades BA.1, BA.5, etc), emerged with a multitude of mutations in the spike protein, posing challenges to existing immunity and vaccine efficacy. In parallel, a new category of variants, known as 'recombinant variants,' emerged in mid-2022 [10]. These recombinant variants stand out due to their unique genomic structures, which differ from the typical lineages, resulting in recombinant lineages like XBB and XBF. Recent studies in 2023 show that 100% of SARS-CoV-2 circulating strains are Omicron or Omicron-Recombinant variants. Yet, despite their numerous structural disparities, initial findings indicate that these recombinant variants do not demonstrate a notable association with increased transmissibility or heightened disease severity [11-13]. 

The investigations into the complex genomic diversity and mechanisms that shaped SARS-CoV-2’s evolution were pivotal during these four years. Natural selection played a vital role, favoring genetic changes that enhanced viral fitness and transmission, while the immune response triggered recombination events, further complicating the virus’s genetic makeup. This complexity stems from several contributing factors, primarily driven by genetic mutations, including substitutions, deletions, frameshifts, and the emergence of lineages with multiple mutations and genetic recombination. Here, we provide a comprehensive overview of how these mutations have influenced the virus’s evolutionary changes and dynamics. By conducting a genomic analysis of samples collected from six diverse geographic regions, we explore the temporal evolution and distribution of different variants. We explore how the main variants have played a pivotal role in shaping the global spread of the virus. Finally, we conduct an in-depth exploration of the genomic landscape of samples collected during the more recent phase of the pandemic, specifically from 2022 onwards, shedding light on the latest developments in SARS-CoV-2 evolution and offering insights into its future trajectory.

## Materials and methods

Data collection

A total of 16,575 SARS-CoV-2 full-length genomes were collected from the Global Initiative on Sharing All Flu Data (GISAID) EpiCov™ (update: October 15, 2023), covering six geographic areas and distributed across 214 countries [14,15]. The metadata associated with the analyzed sequences is available on GISAID up to January 28, 2023, via epicov.org/epi3/epi_set/240201aw.

These genomes, obtained from samples collected between December 2019 and October 2023, were selected based on genome completeness (genome length more than 29,000) and coverage (less than 1% undefined bases and fewer than 0.05% unique amino acid mutations). Additionally, only sequences with fully documented collection dates were considered. A sample randomization process was conducted to eliminate bias and ensure well-distributed representation across different time periods and geographic regions. To account for differences in sample sizes, the prevalence of specific lineages or mutations was calculated by dividing their count by the total number of samples within a given time duration and/or region. The list of genomes and their metadata is provided in Appendix 1 and visualized in Appendix 2.

Genomic data analysis

Each of the retrieved sequences was aligned against the SARS-CoV-2 reference sequence (Wuhan-Hu-1/2019) using the MAFFT command line (version 7). The sequences were then processed using VCFtools (version 4.1) for the identification of variants by analyzing variant call format (VCF) files, and SnpEff (version 5.1d) to annotate the identified mutations and variants [16,17]. We relied on the International DataBase for Sars-CoV-2 Variant (IDBSV) (version October 2023) as a supplementary source for variant annotation and effect prediction [18].

For the phylogenetic classification of the sequences, we utilized the Augur pipeline (version 23.1) for the construction of phylogenetic trees and the analysis of evolutionary relationships among the virus samples [19]. To interpret the classification of the analyzed samples, we performed based on the Nextclade annotation for Clades (version 2.9.1) and PANGOLIN annotation for lineage (available at cov-lineages.org) [20]. Finally, for the visualization of the phylogenetic trees generated, we utilized the web platform auspice (version 2.42.0) [20].

Comparative analysis

In our analysis, to identify individual mutations, we compared the retrieved samples to the Wuhan-Hu-1/2019 reference genome. Initially, samples were organized into timeframes, categorized by the year or month of collection, to trace the virus's genetic changes over time. Then, these samples were categorized by their geographical region of identification (continents) to assess global genetic diversity. Cases that deviated significantly from typical patterns were highlighted at the country level, providing a granular view of unusual mutations or outbreaks.

Due to the scant number of samples from December 2019 (N=4), these were pooled with those from 2020 to ensure a comprehensive dataset for early pandemic analysis. This strategic organization allowed for the identification of significant mutations and their distribution patterns, offering insights into the virus's adaptation and spread across various global and temporal landscapes.

Mutation analysis

In our study, frequent mutations are defined as the top 10 mutations with the highest abundance observed within every month. We also define hotspot mutations as those present in at least 75% of all samples analyzed across the entire dataset. This dual categorization captures both temporal changes, reflecting fluctuations in mutation prevalence over time, and stable, population-wide genetic trends in SARS-CoV-2 evolution. The genetic evolution rate is estimated using a linear regression model for each year from 2020 to 2023, providing insights into the virus’s adaptation and evolution during specific periods. The results from each year were compared to assess the dynamics of genetic changes over time.

## Results

Mutational landscape

**Figure 1:**
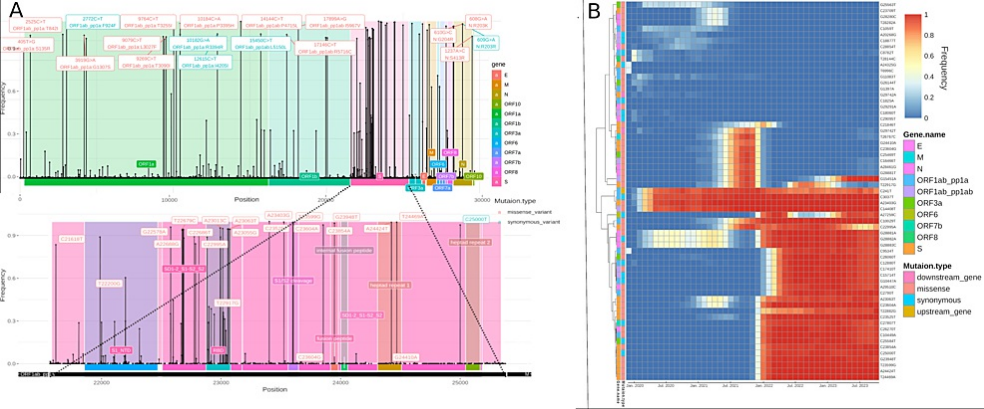
Mutational landscape of SARS-CoV-2 from the period between December 2019 and October 2023. A: Lollipop chart illustrating the distribution of mutations throughout the severe acute respiratory syndrome coronavirus 2 (SARS-CoV-2) genome. The x-axis represents the genomic position, while the y-axis indicates the frequency of each mutation detected. Different colors and annotations identify specific mutation types or regions within the virus’s genome. The bottom panel is a focused examination of mutational frequency, specifically zoomed in on the Spike (S) gene region. B: Heatmap of mutation, where each row corresponds to a specific mutation and each column represents a month in the viral evolution. The annotations on the left side of the heatmap correspond to mutation type and corresponding gene.

A total of 21,580 distinct mutations were identified with varying frequencies across the 16,575 analyzed genomes. We classify these mutations into different types: the majority were missense mutations, accounting for 54.4%, and coding for 4480 distinct amino acid changes in viral proteins. Synonymous mutations represented 41.9%, while insertions, deletions, and nonsense mutations made up the remaining 3.6%. In this section, we highlight the hotspot mutations, with particular emphasis on the changes observed during the four years of evolution. The full dataset of mutations identified, including the date and location of identification and corresponding variants, is provided in Appendix 3. As shown in Figure 1A, the mutations A23403G, C14408T, and C3037T, along with G28881A, G28883C, and G28882A, were the most prevalent. We observed occurrences of mutations in the RBD of the spike protein, including C22995A (T478K) and A23063T (N501Y), crucial for ACE2 receptor interactions, at 61.06% and 55.33% respectively, along with C23604A (P681H) at the S1/S2 cleavage site at 56.74%, C23525T (H655Y) in the fusion peptide region, G21987A (G142D) in the N-terminal domain (NTD), and mutations in the heptad repeat regions T24469A (N969K) and T23599G (N679K) in 52% of samples. Other notable mutations included A24424T (Q954H) in the HR2 region, G23948T (D796Y) in the internal fusion peptide, and several RBD mutations such as G22992A (S477N), G22578A (G339D), and A23013C (E484A), with frequencies ranging from 47.50% to 50.17%, highlighting the evolving mutational profile of the spike protein. During the 46 months of our study, significant variability in the frequency and distribution of these mutations was observed. Our analysis indicates that certain mutations, like the spike A23403G (D614G), quickly achieved global dominance, while others, such as the spike T22918G (L452R), exhibited a more sporadic distribution, leading to concentrated outbreaks in specific regions.

The heatmap, in Figure 1B, provides a chronological gradient of the top 10 most frequent mutations for each month throughout the study period (total N=63). Notably, the mutations were divided into two main clusters. The first cluster (A) encapsulates a first clade (A.1) of mutations such as A23403G, C14408T, and C3037T that consistently demonstrated high frequencies across multiple time points since the first months of the pandemic. The second clade (A.2) encapsulates 22 mutations that emerged progressively and attained high frequency later during the second half of the pandemic. Notably, within this group, the paired mutations C26060T and C29510C stand out, as well as a collective of spike protein mutations (C23604A, T22882G, C23525T, C26270T, C10449A, C23854A, G23948T, T23599G, A24424T, and T24469A) that have gained gradual dominance since December of 2021. On the other hand, the second cluster (B) was characterized by mutations with fluctuating frequencies. Subclade B.2 included the mutations T26767C, C16446T, G28881T, C23604G, and C25469T that were highly prevalent from July to November 2021. Otherwise, subclade B.1 included the mutations T22917G and G15451A which showed dominance from July 2022 to February 2023 and from April to October 2023, respectively.

Evolution rate

**Figure 2:**
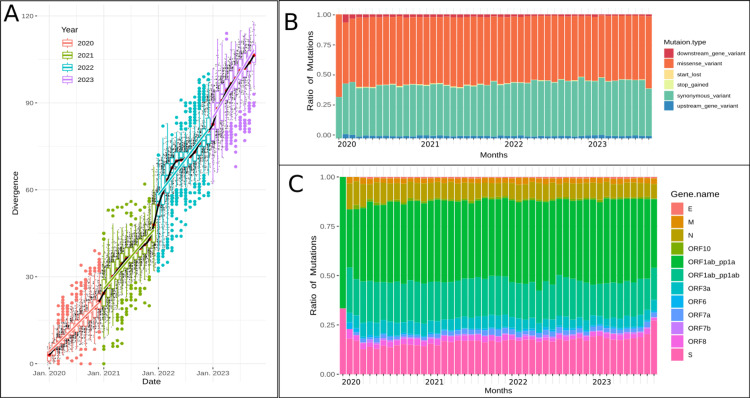
SARS-CoV-2 genetic evolution over time. A: Scatter-plot with a line model showing the divergence of the severe acute respiratory syndrome coronavirus 2 (SARS-CoV-2) over time. The x-axis represents dates, and the y-axis shows the divergence measured as the number of mutations accumulating over time. Each point represents a viral sample color-coded by its year of collection. B: Stacked bar chart of frequency distribution of mutation types. The y-axis shows the ratio of mutations, and the x-axis is segmented by months and years, with each color corresponding to a different type of mutation. C: Stacked bar chart of frequency distribution of mutant regions. The y-axis indicates the ratio of mutations, while the x-axis represents the months and years and the colors represent different genes within the SARS-CoV-2 genome.

Next, to gain a more comprehensive understanding of the evolutionary dynamics, the temporal change in the virus’s genetic evolution speed was explored. Our data analysis showed that, initially, SARS-CoV-2 exhibited a relatively stable genetic composition, suggesting limited variability during the early stages of the pandemic. This initial phase of stability was characterized by a lower rate of genetic changes, indicative of a virus adapting to a new host species. However, as the virus spread globally, the emergence of mutations with enhanced transmissibility led to an acceleration in the virus’s evolutionary rate. The molecular clock analysis, depicted in Figure 2A, estimates this rate at approximately 0.07 substitutions per day (25.55 substitutions per year). Continuous monitoring over the next three years has revealed a significant increasing trend in this rate, aligning with the emergence of more transmissible and potentially immune-evasive variants. Specifically, the evolutionary rate was estimated at 0.051 substitutions per year in 2020, reflecting the initial adaptation phase of the virus in humans. The evolution increased to 0.06 in 2021, coinciding with the emergence of several variants of concern that demonstrated higher transmissibility and some level of immune escape. The rate rose to 0.067 in 2022, and further escalated to 0.086 in 2023. This upward trend is a clear indicator of the virus's ongoing adaptation to the human host and the dynamic nature of the pandemic. Interestingly, a considerable decrease in the intercept of the linear model estimated in the years 2021 and 2022 was observed. This decrease might indicate the introduction of new lineages of the virus with a higher mutation rate by early 2022, suggesting a pivotal moment in the pandemic where the virus began evolving more rapidly. In the following section, we delve deeply into the distinct characteristics of the various lineages of SARS-CoV-2 that have emerged and spread throughout this period and in different geographic regions. Following this, we narrow our focus specifically to the analysis of samples collected after 2022 to provide a more refined understanding of the observed evolutionary shift observed during this time. Detailed statistics about the estimated evolution rate for each month are provided in Appendix 4.

Regarding the changes in the types of identified mutations in the SARS-CoV-2 genome over time, Figure 2B presents a clear and consistent pattern. Specifically, the data showed no significant temporal variation, maintaining a steady distribution of 53.5% missense mutations, 42.09% synonymous mutations, and 2.097% upstream mutations. In contrast, Figure 2C highlights small fluctuations in mutation frequencies across different genes of the virus, notably within the ORF1a and spike protein genes (averaging 48.5% and 15.6%, respectively, exhibiting increases of 3% and 7.1% between January 2020 and September 2023, respectively) reflecting selective pressure on these specific regions of the virus's genome.

Phylogenetic analysis and lineage evolution

**Figure 3:**
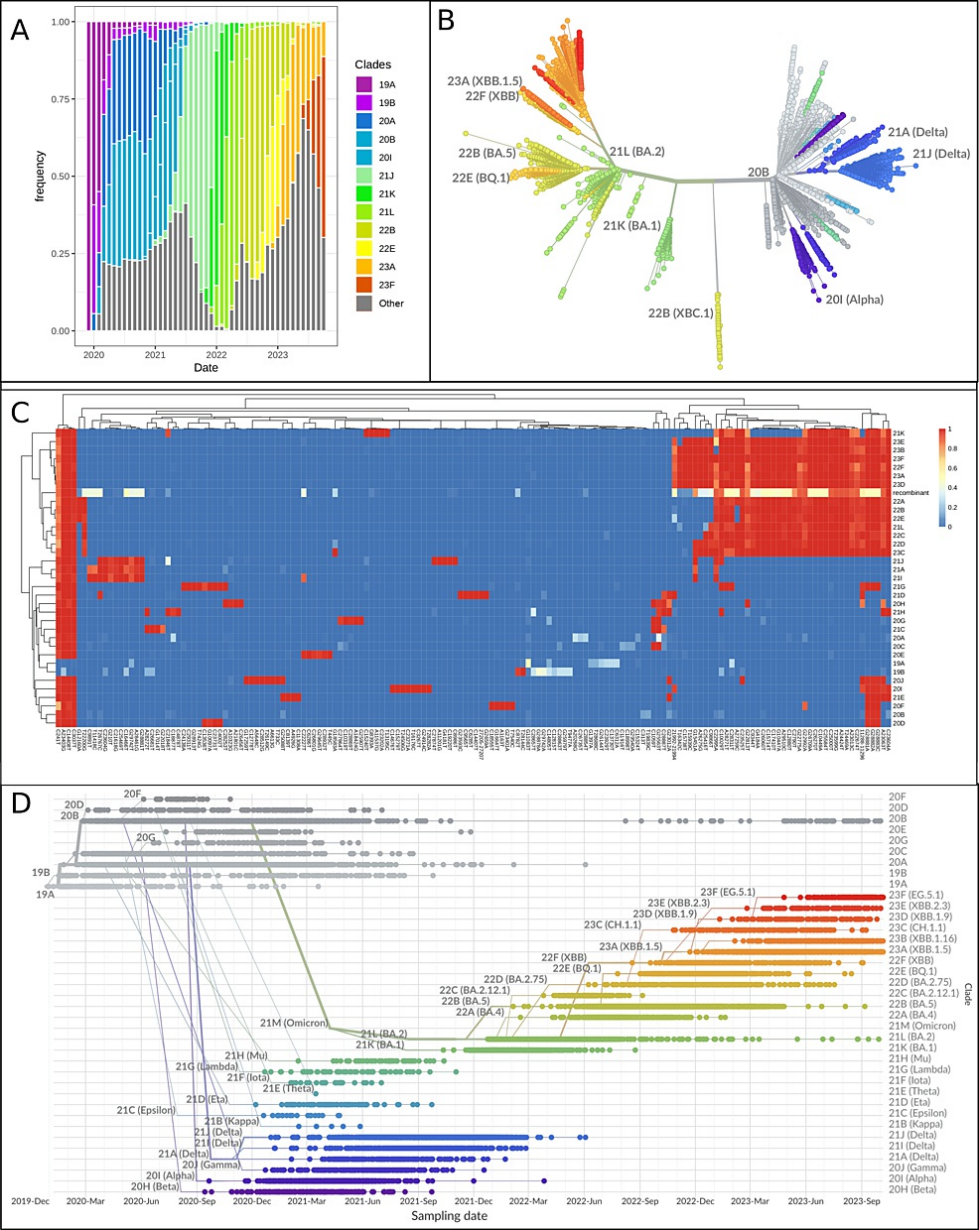
Evolution of SARS-CoV-2 variants over time in the period between December 2019 and October 2023 A: Frequency of severe acute respiratory syndrome coronavirus 2 (SARS-CoV-2) clades over time. The different colors represent different clades, the x-axis represents months and the y-axis shows clade frequency. B: Radial phylogenetic tree representing the evolutionary relationships among different variants or lineages of SARS-CoV-2. The branches are color-coded to differentiate between the clades. C: Heatmap of mutation per SARS-CoV-2 clade. The intensity of color from blue to red indicates the frequency of each mutation in the corresponding clade. D: Evolution trajectory plot represented with chronological phylogenetic tree showing the emergence and prevalence of different SARS-CoV-2 lineages over time.

Throughout four years of the virus’s spread, various new lineages of SARS-CoV-2 have emerged, each characterized by distinct genomic features. As depicted in Figure 3A, the initial phase of the pandemic, starting in December 2019, was marked by the predominance of clade 19A, representing the early strain of the virus. Throughout 2020, clades 20A and 20B emerged and became notably prevalent, with clades 20B and 20A accounting for approximately 40% and 33% of the samples, respectively, by August 2020. The year 2021 witnessed a significant transition in the viral landscape, with the emergence and prevalence of clades 20I (Alpha variant), 21J (Gamma variant), and 21K (Beta variant). The latter half of 2021 saw an increasing dominance of clade 21J (Delta variant), indicating the virus’s ongoing adaptation. By April 2022, clade 21L (Omicron) emerged as the predominant strain, representing nearly 79% of the total. Subsequently, multiple Omicron variants, including clades 22E, 23A, and 23F, alternated in prevalence up to October 2023.

In terms of divergence, the phylogenetic tree in Figure 3B reveals the presence of three main evolutionary branches, each signifying distinct paths of viral evolution. The first branch, starting with 20A, splits into several sub-branches: 20C leading to clades 20H (Beta), 21F (Iota), and 21H (Mu); 21A (Delta) branching into 21J and 21I (both Delta variants); and 20B evolving into 21G (Beta) and 20I (Alpha). The second major branch begins with 21M (Omicron) and evolves into 22A, 22C, and 22B, along with several subclades that emerged after 2022. Finally, the 20B (XBC) lineage forms its own unique branch, branching out separately and distinctly from other lineages.

The analysis of clades based on mutation frequencies revealed distinct profiles in the heatmap (Figure 3C) and phylogenetic tree (Figure 3D). Remarkably, this analysis identified three primary clusters. Similar to the phylogenetic clustering results, the first cluster included clades from the early pandemic phase, such as 20A and 20B, mainly sharing the spike D614G mutation. The second cluster featured three Delta clades (21A, 21I, 21J), closely linked due to shared mutations like T26767C, C23604G, C21618G, and C25469T. The third cluster encompassed all 14 Omicron clades and recombinant lineages, united by mutations like A23013G, C216114T, T22917G, G22992A, C22995A, G23012A, G23032A, and C23064T.

To represent this evolution over time, we depicted the chronological phylogenetic tree of these samples in Figure 3D. In 2020, the early strain, clade 19A, dominated, while in 2021, clades 20I and 21K emerged alongside clade 21A. Moving into 2022, Omicron variants, with clades 21M, 21L, 22B, and 22E, gained prominence. By 2023, the phylogenetic landscape diversified further with new clades like 22F, 23A, 23B, and 23F, while clades 22B and 22E remained prevalent, which underscores the dynamic nature of the SARS-CoV-2 virus and its continuous evolution.

Geographic distribution

**Figure 4:**
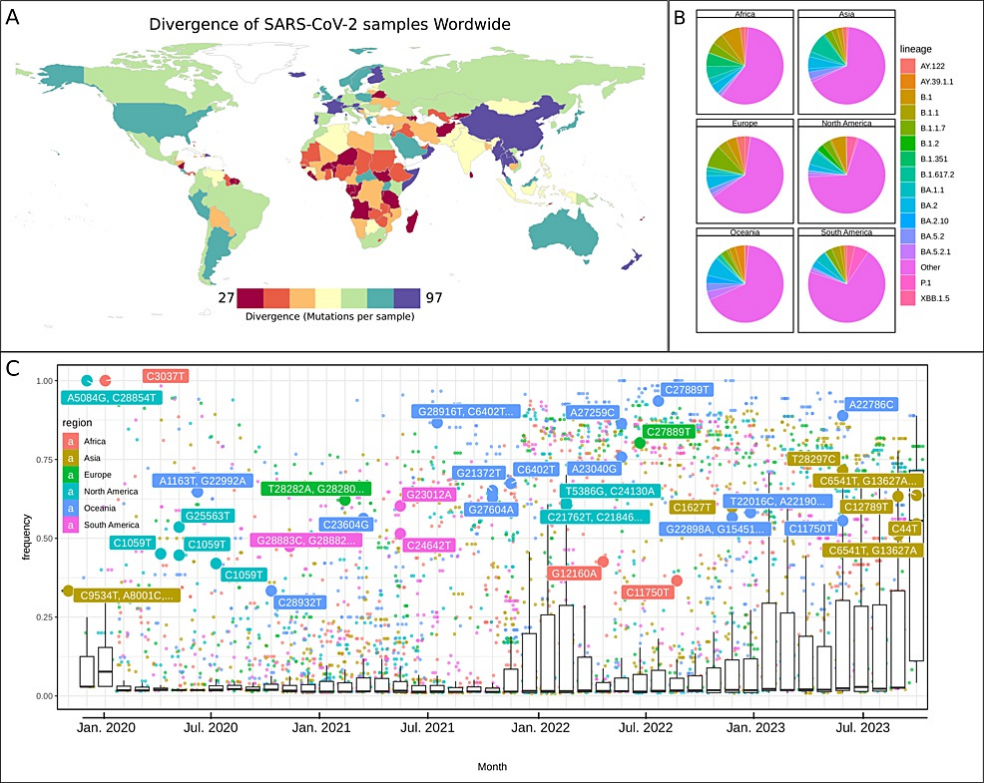
Geographic distribution of SARS-CoV-2 variants in the world. A: World map colored according to the divergence of severe acute respiratory syndrome coronavirus 2 (SARS-CoV-2) samples. B: Pie charts showing the diversity of SARS-CoV-2 lineages in each of the six continents. C: Boxplot and scatterplot showing the frequency of mutations in each of the six continents. Each point represents a mutation, color-coded by the region where it was detected. The box plots below the scatter plot represent the distribution of mutation frequencies over time. Frequent mutations are labeled with their specific nucleotide changes providing an overview of how frequently certain mutations are appearing across the global SARS-CoV-2 samples.

We analyzed variations in the SARS-CoV-2 genomic landscape across global regions. Figure 4A presents a global map that illustrates the estimated genomic divergence rate observed in each country as of October 2023. On average, the collected samples harbored 69 mutations each. However, notable regional disparities were observed between different continents. For instance, Africa showed the lowest median divergence, averaging 55.5 mutations per sample, in contrast to Asia and Oceania, which had the highest median divergence at 69 and 71.5 mutations per sample. On a country-specific level, nations like Guinea-Bissau, Canary Islands, Burkina Faso, Tanzania, and Rwanda reported remarkably lower divergence rates, each with fewer than 25 mutations per sample. Conversely, in Asia, countries such as Brunei, Marshall Islands, Myanmar, China, and Laos, along with certain islands including Reunion, Dominican Republic, Palau, and Micronesia, displayed notably higher divergence rates, exceeding 70 mutations per sample on average. The observed disparities stem from the introduction and subsequent spread of different variants in different regions, as illustrated in Figure 4B. While BA.1.1 was the most prevalent form of the virus in Asia, South America and North America, this lineage was less frequent in Africa, Europe and Oceania where lineages B.1, B.1.1.7 and BA.2 were the dominant.

Actually, by late 2020, the Alpha variant (Lineage B.1.1.7) quickly became dominant globally, while the Beta variant (Lineage B.1.351) from South Africa had a more limited spread. Specific mutations were prevalent in Europe in the second quarter of 2021, such as C5388A (ORF1:A1708D) and C23709T (S:T716I), detected in around 70% of samples-higher than the global average of 32%. In South America, C23525T (S:H655Y) was detected in 48.54% of samples, deviating by 38.07% from the global average. Oceania reported G21372T (ORF1:Q7036H) in 60.33% of samples by the fourth quarter of 2021, 44.58% above the global average. By the first quarter of 2023, Asia registered A28330G (N:G19G) and C1627T (ORF1:L454L) in roughly 57% of samples, significantly exceeding the global average of 13.96%. In the same period, Oceania observed five mutations, including T22016C (S:W152R) and G22331A (S:G257S), at a frequency of 54.90%, well above the global average of 15%. Figure 4C summarize the frequency of the most significant mutations occurring each month across the six continents.

Geographic trends were also evident in SARS-CoV-2 lineages. In Europe, the B.1.1.7 lineage rose to 67% of samples by the second quarter of 2021. Asia saw the B.1.617.2 lineage at 42%, higher than the international average. The P.1 lineage in South America reached nearly 40%, surpassing the regional average during the same period. North America exhibited distinct lineage shifts in the first quarter of 2022, with BA.1.1 dominant at over 53%. The following year, the XBB.1.5 lineage was detected in 32% of the region's samples, double the global average. By the last quarter of 2023, while recombinant lineages emerged globally, the XBB.1.5 lineage remained prevalent in North America at 31.44%. South America also recorded a higher frequency of this lineage at 27.54%, exceeding the global average. Asia and Africa noted the XBB.1.16 and XBB.1.17.1 lineages at frequencies surpassing their worldwide averages. Figures 3B, 3D depict distinctive mutations for specific regions, with detailed mutation frequencies per region in each month documented in Appendix 5.

Genomic landscape since the emergence of Omicron 

**Figure 5:**
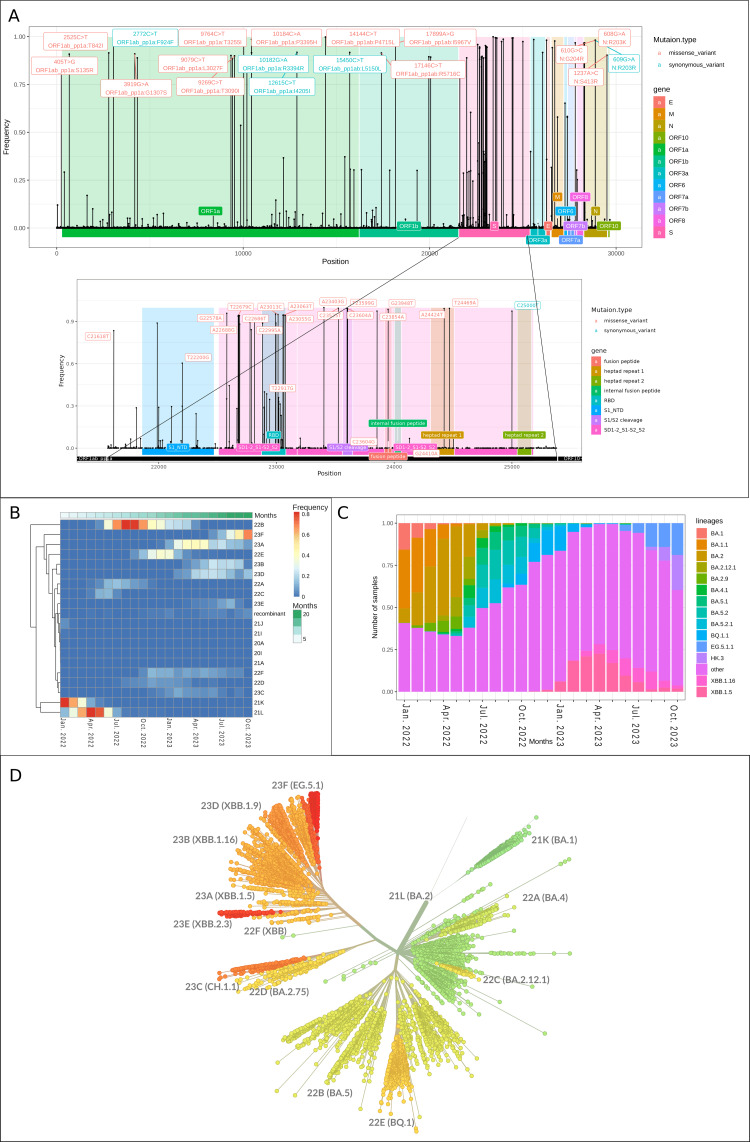
SARS-CoV-2 genetic evolution during the period between January 2022 and October 2023. A: Lollipop chart illustrating the distribution of mutations from the samples collected between January 2022 and October 2023. B: Heatmap showing the frequency of different clades over time, with the intensity of color indicating the level of similarity or difference. C: Stacked barplot showing frequency of severe acute respiratory syndrome coronavirus 2 (SARS-CoV-2) clades. Bars are color-coded by SARS-CoV-2 clades. D: Unrooted phylogenetic tree representing the evolutionary relationships among different variants or lineages of SARS-CoV-2.

Ultimately, our investigation focused on samples collected after January 2022 to delve into the evolving landscape of SARS-CoV-2 variants, particularly highlighting the impact of the Omicron variant's emergence. We observed a more homogeneous mutation profile, indicative of Omicron's higher mutation rate compared to earlier variants. This variant, marked by widespread dominance, is associated with 40 hotspot mutations-each with a frequency exceeding 75%, as shown in Figure 5A. Notably, 21 of these are missense mutations in the spike protein region, emphasizing the gene's adaptive evolution. The dynamics of SARS-CoV-2 clades and lineages from 2022 to 2023 are depicted in the heatmap (Figure 5B) and bar plot (Figure 5C). Clades 21K (BA.1) and 21L (BA.2) initially dominated early 2022, followed by the significant emergence of clade 22B (BA.5) in June. Clades 22E (BQ.1), 23A (XBB.1.5), and 23F (EG.5.1) succeeded in subsequent months, underscoring the variants' dynamic nature.

Phylogenetically, Figure 5D classifies Omicron samples into two primary clades that can be identified as BA lineages and XBB lineages. Within the BA cluster, two main sublineages emerged: BA.2 (clade 21L) and BA.5 (clade 22B). Clade 21L is characterized by mutations such as S:T19I and S:S371F. Clade 22B, which evolved from 21L, acquired additional mutations that led to the development of BQ.1. This clade is distinguished by specific mutations, including S:D405N and ORF1a:L3829F. Additionally, clade 23C (lineage CH.1.1), a more recent addition to the BA cluster, showcases a unique genetic profile with mutations like S:R408S, and notably lacks the S:K417N mutation found in BA.2. In contrast, the XBB cluster originated as a recombinant variant, combining lineages 21L and 22D. This recombination resulted in the XBB cluster acquiring unique mutations, such as S:V445P. Within this cluster, the sub-clade 23B (XBB.1.16) developed mutations like S:E180V, while 23A (XBB.1.5) features mutations that affect ACE2 receptor interaction. Another sub-clade, 23F (EG.5.1), is identified by its distinct mutations, including S:P26- and S:F486P, further contributing to the genetic diversity within the XBB cluster.

## Discussion

Our study explores the dynamic landscape of SARS-CoV-2 evolution and adaptation, shedding light on how mutations, particularly in the spike protein and the RBD, promote the virus’s continuous efforts to optimize its genetic makeup. Throughout our analysis of samples spanning four years, these mutations have influenced several aspects of the virus, including transmissibility, immune evasion, and pathogenicity. The rapid emergence of variants of concern, such as Omicron, characterized by a heightened mutation rate and numerous hotspot mutations, underscores the virus’s adaptability. Understanding the functional consequences of these mutations is pivotal for predicting the virus’s behavior and developing effective countermeasures.

Over this four-year period, we observed a significant increase in the evolutionary rate of the virus, with notable shifts in its genetic makeup. Initially, SARS-CoV-2 exhibited a relatively stable genetic profile, but the emergence of mutations associated with increased transmissibility led to an acceleration in its evolutionary rate. The evolutionary rate of SARS-CoV-2, initially observed at 0.05 substitutions per day in 2020, demonstrated an increasing trend over time. By 2023, this rate escalated to approximately 0.086 substitutions per day which aligns with findings from other studies conducted during the same timeframe, emphasizing the virus’s substantial adaptation capacity [21,22]. The higher evolutionary rate signifies the virus’s ability to rapidly generate genetic diversity, potentially contributing to its continued persistence and adaptability to changing environmental conditions. However, the noticeable decline in the intercept of the linear model during 2022 indicates the emergence of new viral lineages characterized by elevated mutation rates during this period. This observation inspired us to perform the analysis with a focus on samples collected after the shift in the evolutionary speed of SARS-CoV-2 during the last two years [21,23].

The analysis of SARS-CoV-2 mutation types reveals a steady distribution of mutation types, with missense and synonymous mutations being the most prevalent. Yet, although insertions, deletions, and nonsense mutations are less frequent, they can have significant functional consequences and should not be overlooked, as highlighted by other studies [18,24]. Remarkably, we observed that SARS-CoV-2 mutations can be classified into two distinct categories: persistent mutations and temporal mutations. Persistent mutations, characterized by their sustained high prevalence over time, suggest specific regions of the viral genome that are subjected to strong positive selection. For instance, the spike mutation A23403G (S:D614G) rapidly achieved global dominance, indicating its potential advantages for the virus in terms of factors like transmissibility and immune evasion. In contrast, temporal mutations are those that have exhibited fluctuating frequencies, indicating a more intricate interplay of selective pressures. An example of such a mutation is the spike T22918G (S:L452R), which displayed a sporadic distribution, leading to localized outbreaks [25]. This observation underscores that certain mutations may not confer a widespread advantage but can instead result in concentrated outbreaks in specific regions, as supported by prior research studies [24,26,27].

Geographically, we observed minor differences in mutation frequencies across regions. These discrepancies can be attributed to various factors, including international travel patterns, interventions by public health authorities, and levels of population immunity. Notably, certain variants, like the Alpha variant, experienced widespread dissemination and established dominance in multiple geographical areas. Conversely, the Beta variant exhibited a more constrained global spread but played a significant role in localized outbreaks in specific regions. The higher prevalence of certain mutations in particular locales, such as C5388A in Europe and C23525T in South America, is likely a consequence of distinct regional influences and dynamics [28]. Our phylogenetic analysis categorized SARS-CoV-2 samples into three main evolutionary branches, each representing distinct paths of viral evolution. These branches are: Early Clades, which primarily include the early SARS-CoV-2 clades such as 19B and 20B; the Omicron and its variants cluster, which stands out due to their distinct genetic profiles and highly mutated genomes; and the lineage XBC and its descendants, forming a distinct branch of their own resulting from the recombination of Delta and Omicron variants. Notably, XBC strains have shown greater sensitivity to serum neutralization compared to XBB variants. However, further investigations are needed to fully understand this cluster of lineages.

In conclusion, the observed shift in the evolutionary speed of SARS-CoV-2, presented in Figure 2A, aligns with the emergence of the Omicron variant, forming its own cluster in the phylogenetic clustering in Figure 3B. Hence, we shifted our focus towards samples collected after January 2022. This variant is particularly striking due to the presence of an astounding 40 hotspot mutations, of which 21 are concentrated missense mutations within the spike protein region [29]. The distribution of SARS-CoV-2 descendent lineages is visually depicted in Figure 5. Initially, clades 21K (BA.1) and 21L (BA.2) were dominant in early 2022. However, a significant transition occurred in June 2022 with the emergence of clade 22B (BA.5). Subsequently, clade 22E (BQ.1) gained prominence, followed by clades 23A (XBB.1.5) and 23F (EG.5.1) in subsequent months, underscoring the dynamic landscape of SARS-CoV-2 variants during this period. Phylogenetically, our analysis reveals two primary clades within the Omicron variant: the BA clusters and XBB clusters, each exhibiting distinct genetic makeups and contributing significantly to the diversity of the Omicron variant [12-13,30].

Finally, based on the prevailing trends and mutational profiles of the observed lineages, we have identified potential clades that could have a significant impact on shaping the future landscape of SARS-CoV-2 and warrant further investigation. Although the clade XBC variant is not yet widely prevalent, it possesses a unique combination of mutations that could enable it to exploit ecological niches left by other variants if it acquires more mutations. 23B (XBB.1.16), as a sub-lineage within the XBB cluster, has exhibited a remarkable capacity for rapid spread, indicating the possibility of it becoming more prominent. The mutations it harbors could lead to changes in the virus's behavior, potentially posing challenges to current public health measures. Given its existing prevalence, there is also a concern that it might further evolve into more virulent forms if it accumulates additional mutations. The prevalence of EG.5.1 (23F) and HK.3 (23H) has notably surged since the summer of 2023, standing out due to their distinct mutation profile. This clade holds the potential to rise to dominance owing to its genetic uniqueness compared to other clades.

While this study provides comprehensive insights into the temporal dynamics and genomic landscape of SARS-CoV-2 evolution, it is important to acknowledge several limitations. Firstly, we noticed a notable lag between the collection and submission dates of the SARS-CoV-2 sequences especially in developing countries, potentially leading to a delay in capturing the latest viral evolution trends. Secondly, our analysis groups samples by continent, which may obscure specific variations at a country level. Such regional aggregation could mask localized viral dynamics and their response to public health measures. Additionally, while the study focuses on the identification of mutations, a detailed structural analysis of these mutations and their impact was not conducted, which is crucial for a complete understanding of their effects on the virus's properties.

## Conclusions

To sum up, our analysis of SARS-CoV-2 evolution over four years reveals several noteworthy findings. We have categorized mutations into two distinct types: persistent, which remain prevalent over time, and temporal, exhibiting fluctuating frequencies. Our phylogenetic analysis has illuminated three primary evolutionary branches, each one representing unique paths in viral evolution. Surprisingly, our research indicates minimal geographic variations, with local lineages disappearing swiftly. Since 2022, the dominance of Omicron and recombinant variants has become evident, signifying a substantial shift in the viral landscape. Looking forward, our study suggests that future dominant strains are likely to emerge from recombinant lineages, emphasizing the need for continuous genomic surveillance, global data sharing, and collaborative research endeavors. As we navigate the ongoing challenges posed by SARS-CoV-2, it is imperative to remain vigilant and adaptable, keeping pace with the dynamic evolution of the virus. Preparedness and cooperation will continue to be essential in our ongoing efforts to combat this ever-changing threat to global health.

## Figures and Tables

**Figure 6:**
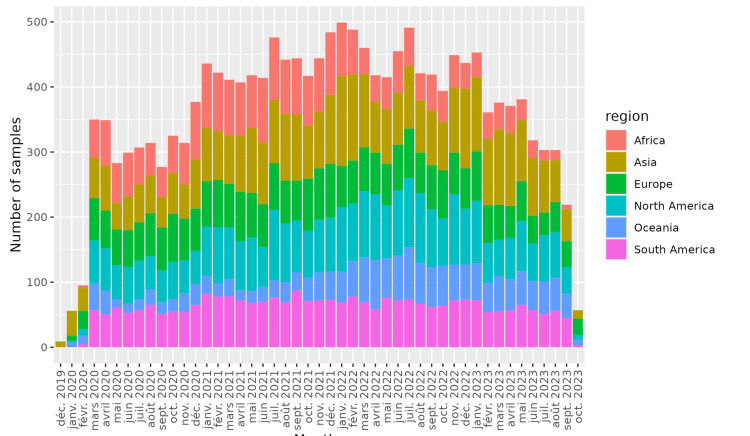
Temporal and geographic distribution of collected samples The stacked bar chart distribution of samples over a time series from December 2019 to October 2023. Each bar represents a month, and within each bar, there are different colors that correspond to different regions. The regions, as indicated in the legend on the right, are Africa (orange), Asia (green), Europe (yellow), North America (blue), Oceania (light blue), and South America (pink).

## Appendices

Appendix 1

List of samples included in the analysis and their metadata:

https://doi.org/10.6084/m9.figshare.24798780.v1

Appendix 2

Appendix 3

List of mutations identified, their positions, date of location of identification in our dataset and their frequency:

https://doi.org/10.6084/m9.figshare.25055045

Appendix 4

Detailed statistics about the estimated evolution rate in each month:

https://doi.org/10.6084/m9.figshare.24798831.v2

Appendix 5

Mutation frequency of SARS-CoV-2 sequences in different continents:

https://doi.org/10.6084/m9.figshare.24798873
